# Supplementary material for: Dynamic Regulation of brsk2 in the Social and Motor Development of Zebrafish: A Developmental Behavior Analysis
Source: Int J Mol Sci. 2023 Nov 19;24(22):16506. doi: 10.3390/ijms242216506 (PMC10671324; doi:10.3390/ijms242216506)
Supplement: Supplementary file 1 [file ijms-24-16506-s001.zip › ijms-2612029-supplementary.pdf]

Supplementary materials

Dynamic regulation of *brsk2* in the social and motor development of zebrafish: A developmental behavior analysis

Jingxin Deng [1#], Chunxue Liu [1#*], Meixin Hu [1], Chunchun Hu [1], Jia Lin [2], Qiang Li [2] and Xiu Xu [1*]

[1] Division of Child Health Care, Children's Hospital of Fudan University, National Children's Medical Center, 399 Wanyuan Road, Shanghai 201102, China

[2] Center for Translational Medicine, Institute of Pediatrics, Shanghai Key Laboratory of Birth Defect, Children's Hospital of Fudan University, National Children's Medical Center, 399 Wanyuan Road, Shanghai 201102, China

[#] Contribute equally

* Correspondence: Xiu Xu: xuxiu@shmu.edu.cn; Chunxue Liu: liuchunxue@fudan.edu.cn

Jingxin Deng: 21111240005@m.fudan.edu.cn

Chunxue Liu: liuchunxue@fudan.edu.cn

Meixin Hu: 22111240011@m.fudan.edu.cn

Chunchun Hu: 14211240007@fudan.edu.cn

Jia Lin: linjiaalyssa@163.com

Qiang Li: liq@fudan.edu.cn

Xiu Xu: xuxiu@shmu.edu.cn

This file contains Supplementary Table 1, Supplementary Figure 1-3 and Legends

Supplementary Table S1. gRNA gene-target sequences, primers for PCR genotyping and qRT-PCR probes used in this study

Item	Oligo name	Sequence (5'-3')	Amplicon length(bp)	gene ID
**gRNA target**				
brsk2a	*brsk2a*-gRNA-E9	GGAGCAGCCGGTACCCAGGA		NC_007136.7
brsk2b	*brsk2b*-gRNA-E4	GGGCAGGTTAACACCCAAAG		NC_007118.7
**PCR genotyping**				
brsk2a	*brsk2a*-gRNA-I9-PCR-F	GAAATAAATGTAGGTTGGGACTAGTGG	345	NC_007136.7
	brsk2a-gRNA-I10-PCR-R	CACACTCCAAACATTAGCCATTTAC		
brsk2b	*brsk2b*-gRNA-I3-PCR-F	GCAGTAGTTCTACCATGGGATTTC	236	NC_007118.7
	brsk2b-gRNA-I4-PCR-R	AGGCTTCTGACTCACTGAGATTTG		
**qRT-PCR**				
brsk2a	*brsk2a*-qPCR-F	GCTGGGTATCGAGAGAAAGGCATG	195	XM_017354251.2
	brsk2a-qPCR-R	AGGAAACTGCAAGTTCCCACTCAAGA		
brsk2b	*brsk2b*-qPCR-F	TCTCCACTTTTGACGAGGCA	131	XM_009303428.2
	brsk2b-qPCR-F	TTACTCTGCCCGTGTTGTGC		

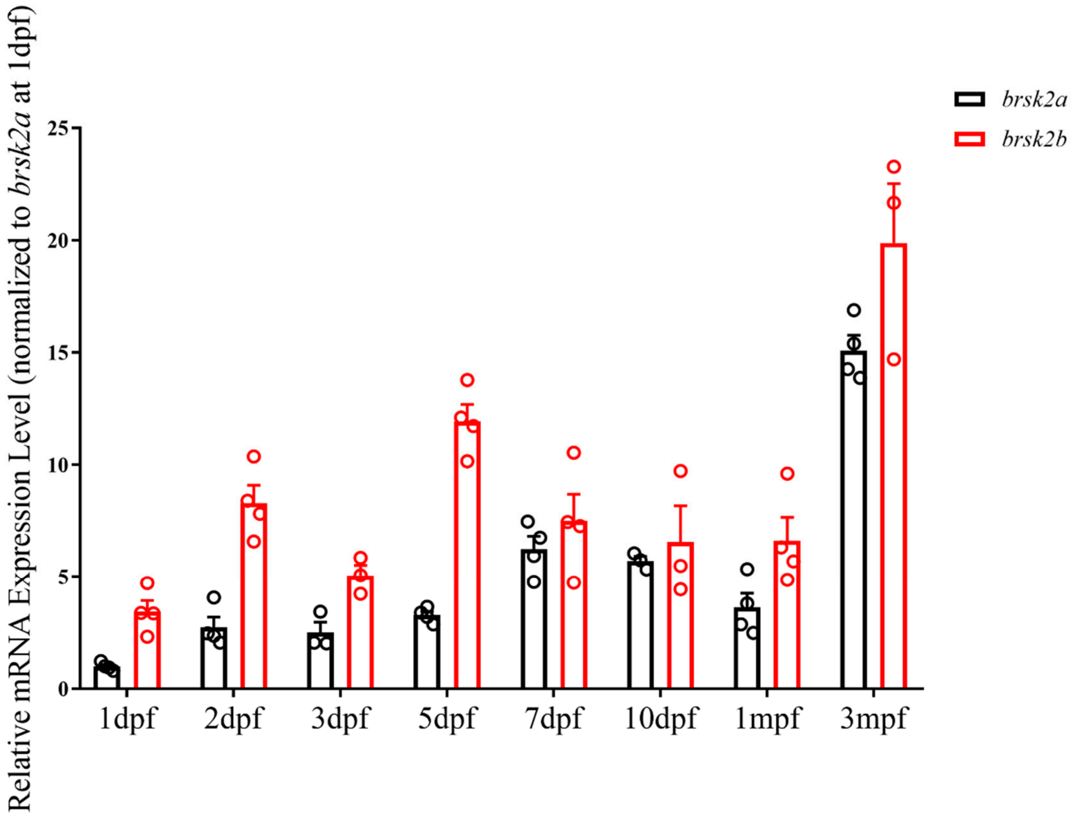

Supplementary Figure S1 Temporal pattern of mRNA expression of zebrafish *brsk2a* and *brsk2b* with individual values at 8 stages.

Data are shown as mean ± SEM, n = 3 for each genotype.

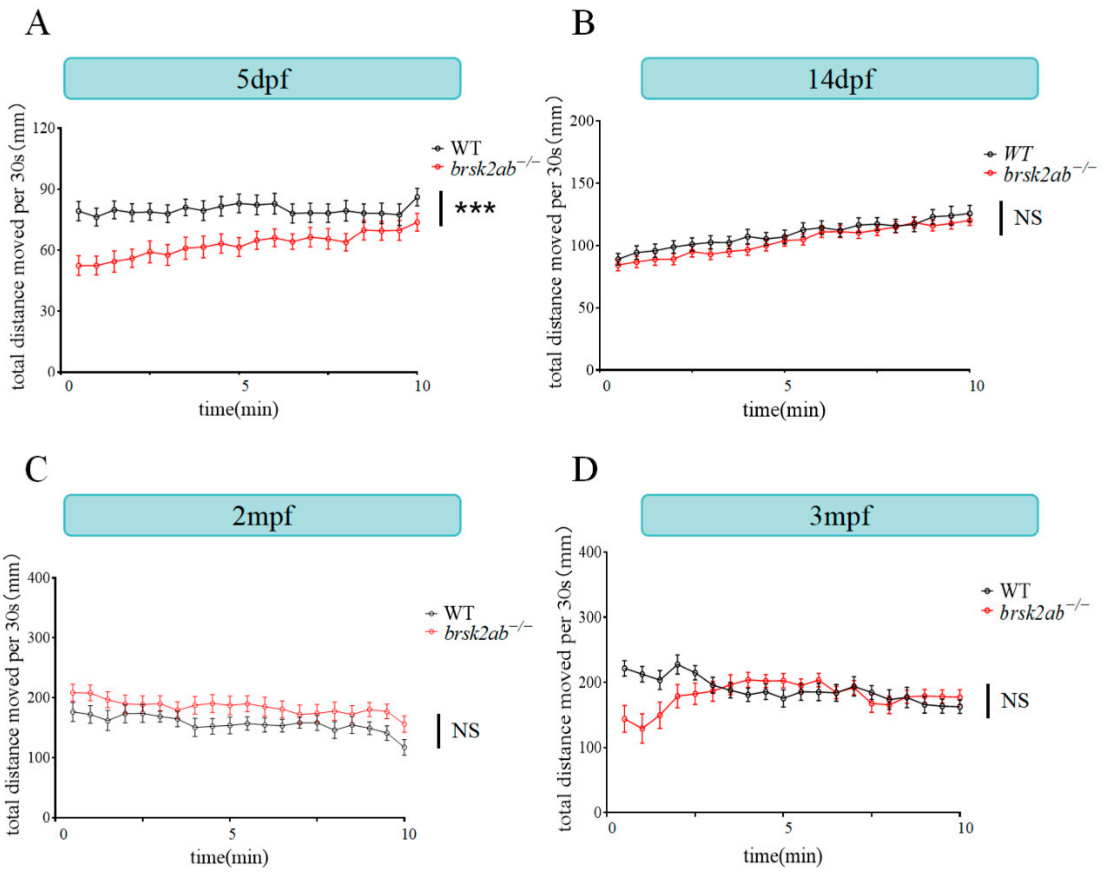

Supplementary Figure S2 The locomotor activity of *brsk2ab*$^{-/-}$ zebrafish over time in different stages.

WT vs. *brsk2ab*$^{-/-}$, 5dpf: n = 48: 48; 14dpf: n = 89: 91; 1mpf: n = 18: 18; 2mpf: n = 26: 20; 3mpf: n = 25: 25. Data are shown as mean ± SEM, ****p* < 0.001.

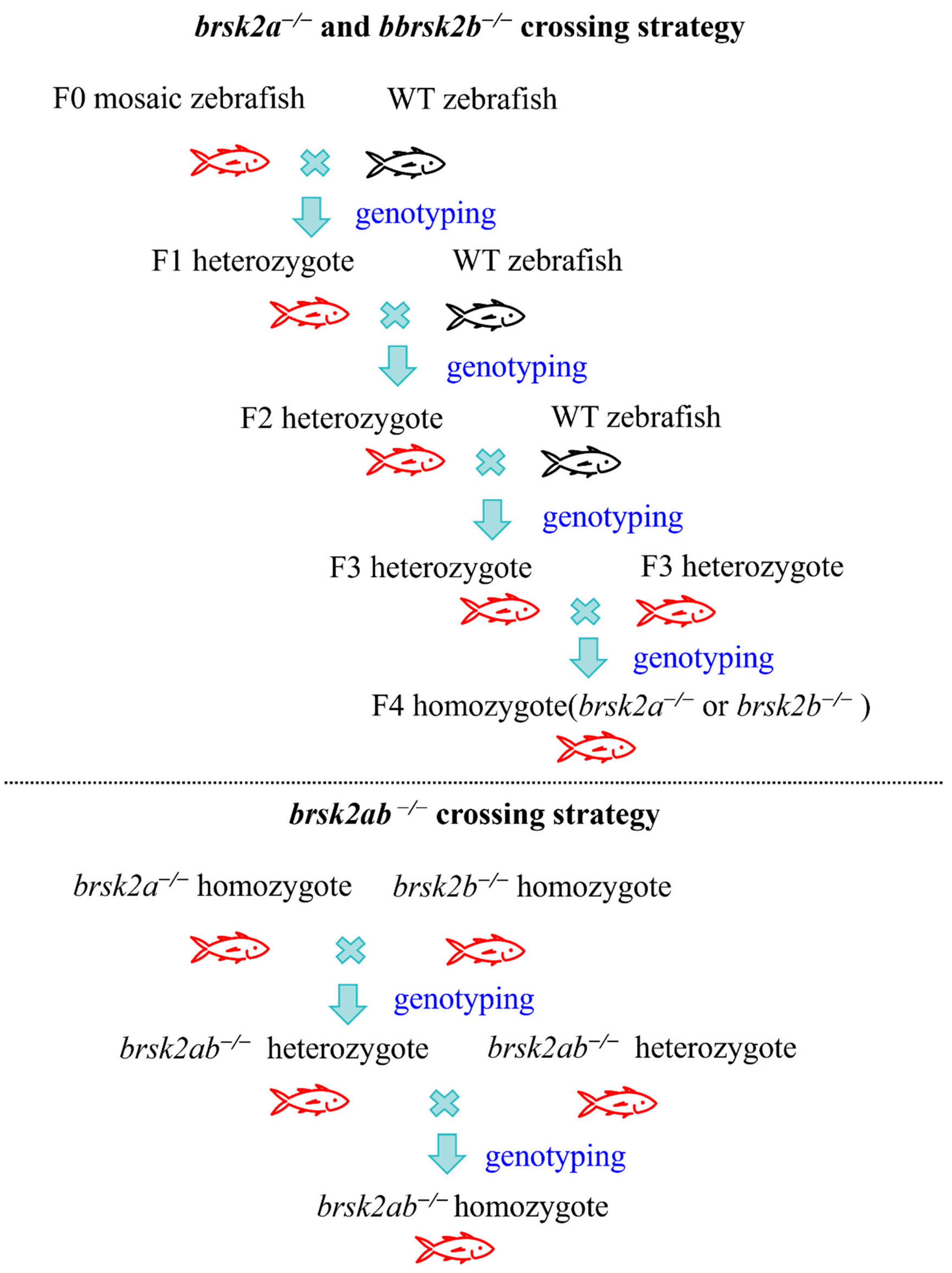

Supplementary Figure S3 Crossing strategy in this study
